# Comprehensive evaluation of SNP identification with the Restriction Enzyme-based Reduced Representation Library (RRL) method

**DOI:** 10.1186/1471-2164-13-77

**Published:** 2012-02-16

**Authors:** Ye Du, Hui Jiang, Ying Chen, Cong Li, Meiru Zhao, Jinghua Wu, Yong Qiu, Qibin Li, Xiuqing Zhang

**Affiliations:** 1 BGI_shenzhen, Shenzhen 518000, China; 2 Department of Clinical Laboratory, Zhongnan Hospital of Wuhan University, 430071, China

## Abstract

**Background:**

Restriction Enzyme-based Reduced Representation Library (RRL) method represents a relatively feasible and flexible strategy used for Single Nucleotide Polymorphism (SNP) identification in different species. It has remarkable advantage of reducing the complexity of the genome by orders of magnitude. However, comprehensive evaluation for actual efficacy of SNP identification by this method is still unavailable.

**Results:**

In order to evaluate the efficacy of Restriction Enzyme-based RRL method, we selected *Tsp *45I enzyme which covers 266 Mb flanking region of the enzyme recognition site according to *in silico *simulation on human reference genome, then we sequenced YH RRL after *Tsp *45I treatment and obtained reads of which 80.8% were mapped to target region with an 20-fold average coverage, about 96.8% of target region was covered by at least one read and 257 K SNPs were identified in the region using SOAPsnp software.

Compared with whole genome resequencing data, we observed false discovery rate (FDR) of 13.95% and false negative rate (FNR) of 25.90%. The concordance rate of homozygote loci was over 99.8%, but that of heterozygote were only 92.56%. Repeat sequences and bases quality were proved to have a great effect on the accuracy of SNP calling, SNPs in recognition sites contributed evidently to the high FNR and the low concordance rate of heterozygote. Our results indicated that repeat masking and high stringent filter criteria could significantly decrease both FDR and FNR.

**Conclusions:**

This study demonstrates that Restriction Enzyme-based RRL method was effective for SNP identification. The results highlight the important role of bias and the method-derived defects represented in this method and emphasize the special attentions noteworthy.

## Background

SNPs are the most abundant markers across the genome. Their relatively uniform distribution and high density make them ideal markers in genome wide association studies (GWAS), comparative or evolutionary genomics study and marker-assisted molecular breeding research. Due to the outcome of HapMap Project [1,2] and the proceeding of 1000-genomes Project [3], over 30 M SNPs in human genome have been genotyped and reported in the dbSNP database [4].

Several genome-wide genotyping technologies have been developed and commercialized, aiming at detecting common SNPs or tagSNPs in parallel [5] (e.g. Illumina BeadArray based on primer extension [6], Affymetrix SNP arrays based on differential hybridization [7] etc.). Although these technologies have obvious advantages such as low costs, whole genome sequencing (WGS) is the most straightforward method for genome-wide identification of SNPs and other types of variants. So far genotyping hundreds to thousands of individuals by WGS is still not affordable for many investigators even considering the dramatic cost decrease due to innovation and update of the technology. Therefore, to fill the gap between current methods, considerable efforts have been made to develop the RRL methods, which have great advantage of reducing the complexity of a genome by orders of magnitude. Recently, lots of RRL strategies have been proposed and proved, such as target enrichment technologies including multiplex PCR, restriction enzyme digestion, selective sequence capture on array [7] or in solution [8], and others (reviewed by Mamanova L [9]).

Compared with other methods, RRL based on restriction enzyme digestion is relatively feasible and flexible, especially for those species without the reference genome [10]. The first RRL using restriction enzyme was described over ten years ago, subsequently many successful cases have been performed in human [11], soybean [12], cattle [13], swine [10] and other species [14]. Recently reported Restriction-site associated DNA (RAD, with similar experimental procedure as RRL) tag method is able to identify and score thousands of genetic markers in the flank region of enzyme recognition sites which randomly distribute across the target genome. RAD method can be widely used in large population studies, enabling not only genotyping and SNP discovery, but also more complex analysis such as quantitative genetic and phylogeographic studies [15-17]. However, RAD method involving multiple steps was labour intensive and typically requires a large volume of starting genomic DNA, moreover, the validation rate was only 85% under high stringency of SNP calling condition in some species like soybean [12]. In a recent study, the researchers utilized RRL of enzyme *Hae III *to identify up to 47 K SNPs with validation rate of 48% in a rainbow trout genome [14]. Although the low validation rate of RAD in soybean and rainbow trout were conferred by polyploidy and a whole genome duplication event related to target genome, the main cause was due to the poor accuracy of the method, indicating the potential possibility of improvement by further optimizing the library building or SNP calling in human beings. Consequently, the low validation rate of RRL method greatly hinders its widespread application. Until now comprehensive evaluation (e.g. power and efficacy of SNP discovery, genome coverage, etc.) is still unavailable, the comprehensive evaluation should be carried out in target genomes with detailed genomic coordinates and available genotyping information. Here we proposed restriction enzyme based RRL construction method and performed comprehensive evaluation for this method, especially for the accuracy of SNPs discovery.

## Methods

### *In silico *digestion and enzyme selection

*In silico *digestion of the human reference genome (http://hgdownload.cse.ucsc.edu/goldenPath/hg18/bigZips) was performed with nine commercially available and methylation-insensitive restriction enzymes. These enzymes were selected upon following criterions: (1) predicted fragments length ranged from 200 to 700 base pairs (bp); (2) proportion of target region overlapped with the repetitive elements; (3) number of putative SNPs in dbSNP database v129 (ftp://ftp.ncbi.nih.gov/snp/organisms/human_9606/) covered by target region. The repetitive elements were determined using RepeatMasker software v3.2.7 (http://hgdownload.cse.ucsc.edu/) [18].

### Library construction and DNA sequencing

**Figure 1:**
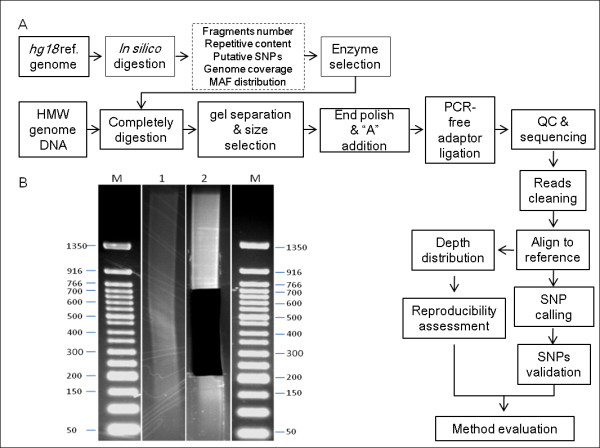
**Main workflow of library construction and data analysis**. (A) The workflow summarized the whole process including enzyme selection, library construction and data analysis. (B) Gel image of completely digestion of YH genome by *Tsp *45I (lane 1) and gel image after gel extraction (lane 2). Lane M shows 50 bp molecular ladder with size indicator aside.

*Tsp *45I RRL was prepared following the main workflow shown in Figure 1A, 5 μg genomic DNA was extracted from peripheral venous blood using QIAamp DNA Blood Mini Kit (Qiagen), then completely digested by *Tsp *45I in 100 μL reaction mixer at 65°C for 1 hour (New England Bio Labs). The digested DNA was separated on a 2% agarose gel and the fragments in the range of 200 bp to 700 bp were excised from gel and purified using QIAquick Gel Extraction Kit (Qiagen). The sticky ends of fragments generated by *Tsp *45I digestion were polished with T4 DNA polymerase and Klenow polymerase, after 'A' base tailing using Klenow exo^-^(3' to 5' exo minus). Then PCR-free adaptors with a single 'T' base overhang at the 3' end were ligated to the above products. The concentration of the libraries was determined by Q-PCR. The libraries were performed 90-cycles paired-end multiplex sequencing on Illumina HiSeq 2000 platform (Illumina).

### SNPs calling and validation

The raw sequencing data were masked from adapter using our own software application Mlinker. Parameters were set up for Mlinker as following: (1) overlapped length between read and adapter was at least 5 bp; (2) minimum of 90% sequence identity between read and adapter; (3) length of reads after adapter-masked was at least 35 bases. The clean reads were mapped to the human reference (*hg18*) using SOAPaligner v2 (http://soap.genomics.org.cn/) with at most 4 mismatches and at least 40 perfectly matched bases.

SOAPsnp v2 [19,20] based on Bayesian model was used to call SNPs on target regions. Five filter steps were used to remove unreliable part of the consensus sequences: (1) both the major and minor alleles should be detected at least twice; (2) the overall sequencing depth, including randomly placed repetitive hits, was less than 200-fold; (3) the copy number of flanking sequences should be less than 2; (4) the genotype quality and average quality score of best nucleotide were at least 20, and average quality score of second best nucleotide was at least 20 if the genotype was heterozygous; (5) SNPs ought to be at least 5 bp away from each other. After filtering, the number of YH consensus sequence was calculated, and discrepancies between the YH genome and *hg18 *reference genome were considered SNPs.

SNP validation was processed in two aspects including detection of SNP calling accuracy and genotyping accuracy. We used high confident SNPs set previously generated by whole genome sequencing (WGS) to evaluate our SNPs calling results. Novel SNPs identified in our results were defined as false discovery loci, and missing SNPs in our results were defined as false negative. The FDR and FNR indicated the accuracy of SNP calling. To evaluate the genotyping accuracy, we calculated the concordance of these SNPs genotyped by RRL method and Illumina 1 M Duo_v3.B BeadArray.

### Genome coverage and Minor allele frequency (MAF) distribution

To compare with commercially available genotyping platform, we set some parameters including genomic coverage (r^2 ^≥ 0.8), MAF (mean/median), and spacing (mean/median) between markers. Allele frequency and linkage disequilibrium (LD) data for the four HapMap populations were obtained from HapMap database (http://www.hapmap.org, phase 2). The value of genomic coverage was calculated according to the method previously described [21], In brief, we performed the naive estimation of coverage using HapMap release24 as reference set of SNPs, which was defined as *R*. The sets of SNPs covered by Illumina genotyping Beadchip or RRL were represented by *T*. The sets of SNPs located in same LD blocks with T sets of SNPs with r^2 ^≥ 0.8 were represented by *L*. Then the value of genome coverage was calculated as (*L*+*T*)/*R*. Human 1 M Duo_v3.B Beadchip from Illumina which cover about 1.1 M SNPs was selected to perform the comparison.

## Results

### Enzyme selection, RRL construction and sequencing

In this study we screened nine restriction enzymes with human genome *hg18 *as reference, we fragmented the whole genome *in silico *according to the enzyme restriction-site sequence, considering to the fragment size of sequencing platform the target fragments ranging from 200 bp to 700 bp were selected, as large variety of fragment length was not recommended for the cluster generation of the Illumina sequencing platform. To minimize the repetitive content, we calculated the frequency of target region overlapped with repetitive elements, the frequency for all enzymes was about 40% to 50%, which was close to that of whole human genome. Therefore, it is difficult to remove the repeat sequences from the RRL for human being (Table 1). Based on the simulation results, *Tsp *45I that specifically recognize a 5-bp site (GTSAC) with a degeneration base in the middle, in principle could call around 1 M putative SNPs. Since we planned to evaluate the SNP calling efficiency by comparing our RRL method and 1 M Duo_v3.B chip, we selected *Tsp *45I to obtain RRL. *Tsp *45I RRL produced approximately 1.5 million fragments ranging from 200 bp to 700 bp, paired end sequences from the sequenced fragments covered 266 Mb region (8.64% of the whole genome) flanking enzyme recognition sites.

**Table 1:** Summary of *in silico *digestion results

Restriction Enzyme			Fragments (200 -700 bp)				Distance between two adjacent reads		#Putative SNPs^b ^
		
	#Total selected fragments	#Total length of target regions^a^	% Percent of coverage	#Length of repetitive on target regions	%percent of repetitive contents	Mean (Mb)	Median (Mb)	S.D.(Mb)	
*Sac *I	65,734	11,832,120	0.38%	4,642,275	39.24%	22,732	442	131.63	48,250
*Ava *I	69,204	12,456,720	0.40%	5,388,814	43.26%	21,582	368	140.26	59,204
*Hin*d III	114,374	20,587,320	0.67%	8,376,468	40.69%	13,027	425	95.84	79,308
*Pvu *II	194,918	35,085,240	1.14%	12,510,491	35.66%	7,607	379	73.01	137,942
*Sfc *I	442,338	79,620,840	2.59%	33,483,887	42.05%	3,303	348	47.28	319,623
*Dra *I	1,131,481	203,666,580	6.61%	74,549,862	36.60%	1,237	235	29.41	774,892
*Tsp *45I	1,479,019	266,223,420	8.64%	104,133,241	39.12%	926	224	25.64	1,074,049
*Bcc *I	2,419,310	435,475,800	14.14%	216,911,945	49.81%	531	170	19.95	1,750,903
*Mbo *II	3,308,660	595,558,800	19.33%	251,315,078	42.20%	365	148	17.04	2,298,087

The *in silico *digestion results of nine restriction enzymes using *hg18 *genome as reference were shown. ^a ^regions sequenced in the final corresponding library and calculated according to pair-end sequencing with average read length of 90 bp. ^b ^The number of putative SNPs are calculated based on dbSNP v129 data.

**Figure 2:**
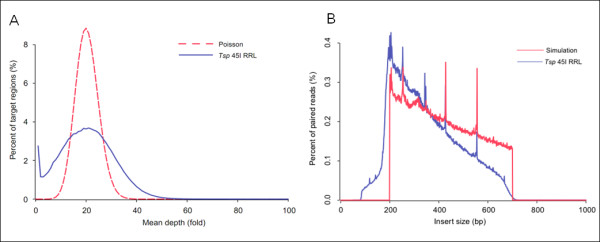
**Insert size and depth distribution of YH *Tsp *45I RRL**. (A) Depth distribution of target region in *Tsp *45I RRL. The red dashed line shows standard Poisson distribution. (B) Insert size distribution in *Tsp *45I RRL. Insert size was calculated based on aligned paired end reads in *Tsp *45I RRL sequencing data. Compared to simulation results, these fragments shorter than 400 bp were over-represented and longer fragments were under-represented. These peaks along the distribution indicated the accumulation of repeat sequence.

The workflow of *Tsp *45I RRL construction was depicted in Figure 1A, which was compatible to Illumina sequencing platform. The genomic DNA was digested with *Tsp *45I entirely and separated by agarose gel electrophoresis (Figure 1B). A total of about 87.38 million reads with an average length of 90 bp were generated in half a lane of Illumina HiSeq 2000 platform, 7.86 G bases were produced (Table 2). Using SOAP software package, about 84.7% of total past filter (PF) reads could be mapped to reference genome with unique map rate of 93.2%, and 80.8% mapped reads were located on target region. Of the 266 M target region in simulation results, 255 M (96.8%) was covered by at least one reads with 20-fold mean depth. The total depth distribution of target regions approximately formed a Poisson distribution (Figure 2A).

**Table 2:** Summary of sequencing and alignment results

Total reads	Total bases (Gb)	PF bases (Gb)	Mapped bases (Gb)	On target region (Gb)	Target region with depth ≥ 1 (Mb)	Mean depth	Mismatch rate
87,382,662	7.864	7.848(99.8%)	6.644(84.7%)	5.374(80.8%)	255.34 (95.9%)	20.40	0.33%

To assess the quality of RRL construction and sequencing, we compared the actual insert size distribution calculated according to the paired end information of sequence reads to that of simulation result. Figure 2B indicated that the length distribution of inserted fragments in *Tsp *45I RRL was approximately from 200 to 700 bp. Compared with the simulative curve, the small fragments were over-presented in the final sequencing results, probably indicating the bias in the process of library construction and cluster generation. Furthermore these peaks in the distribution with consistent patterns were contributed by the accumulation of repetitive elements.

### SNP identification and validation

For SNP calling, highly stringent filter criteria (see Methods for detailed information) were used. To evaluate the accuracy of SNP calling, we compared the identified SNPs to YH SNPs data generated by WGS [22] and dbSNP database (Table 3). Of 257,631 SNPs identified by RRL sequencing, 222,028 loci were previously detected by WGS and the validation rate was 86.18%, the remaining 35,603 loci were not involved in WGS result. Of 299,164 SNPs from WGS results which located on the target regions of RRL, 77,136 SNPs were missed in the RRL results. Based on statistic analysis, the false discovery rate (FDR) and false negative rate (FNR) of *Tsp *45I RRL method were 13.82% and 25.78%, respectively.

**Table 3:** Validation of SNP calling

		On target regions of RRL	
		
		SNP	Not SNP
SNP dataset generated by WGS	SNP	222,028	77,136
	Not SNP	35,603	---

SNP calling results generated by RRL sequencing and WGS were compared by calculating the number of loci identified as SNPs by two methods equally or differently regardless of the concordance of genotyping. From the results the false discovery rate of RRL method was about 13.82% (35,603/(222,028+35,603)) and false negative rate was 25.78% (77,136/(77,136+222,028)).

To figure out the main causes, false discovery and false negative loci were selected and calculated for their identification or missing. From Table 4, several evidences were brought forward: first, low depth coverage contributed to about 38.22% of FNR but less of FDR; second, up to 69.79% of false negative loci and 30.67% of false discovery loci were filtered out due to low quality score, indicating great impact of sequence quality on SNP calling; third, the copy number parameter was used for eliminating the effect of repeat sequences on accuracy of SNP calling. Contrary to quality score, up to 70.32% of FDR and 33.17% of FNR were due to copy number filter, suggesting that repeat elements had larger effect on FDR. We calculated the proportion of false discovery and false negative SNPs on repeat regions, the results showed that 84.2% of FDR and 42.5% of FNR were caused by repeat regions, which was consistent with the previous results; finally, the influence of high depth filter could be ignored. Upon the statistics, the major contributors to FDR and FNR were base quality and repeat sequences, this part of FDR and FNR could be optimized by increasing sequencing depth and quality, or masking repeat sequences before SNP calling. In addition, about 78.65% of false discovery SNPs were present in dbSNP database, implicating that they might be novel identification. However, further experimental validation in common populations were necessary to figure out whether this part of false discovery SNPs was novel. Except the above explanation of SNPs with appropriate interpretations, the remainder made up most of veritable FDR and FNR. The estimated FDR and FNR in our *Tsp *45I RRL should be as low as 2.3% and 6.67%, respectively.

**Table 4:** Detailed interpretations for high False Discovery Rate and False Negative rate

False Discovery Rate (FDR) class		
**Intersection of the reasons**	**Number of loci (percentage)**	**In dbSNP v129**

Reasonable interpretations for SNPs filtered out in YH WGS results	29,687(83.38%)	23,348(78.65%)

1. Low depth (<2)	394(1.11%)	---
2. Low quality (< 20)	10,920(30.67%)	---
3. High copy number (> 2)	25,036(70.32%)	---
4. High depth (> 200)	538(1.51%)	---

Overcalled for unknown reasons in RRL sequencing	5,916(16.61%)	615(10.40%)

False Negative rate (FNR) class		

Intersection of the reasons	Number of loci (percentage)	In dbSNP v129

Reasonable interpretations for SNPs filtered out in RRL sequencing results	57,169(74.11%)	45,216(79.09%)

1. Low depth (<2)	29,478(38.22%)	---
2. Low quality (< 20)	53,830(69.79%)	---
3. High copy number (> 2)	25,587(33.17%)	---
4. High depth (> 200)	43(0.06%)	---

Allele dropout in RRL sequencing	19,967(25.89%)	10,724(53.71%)

**Figure 3:**
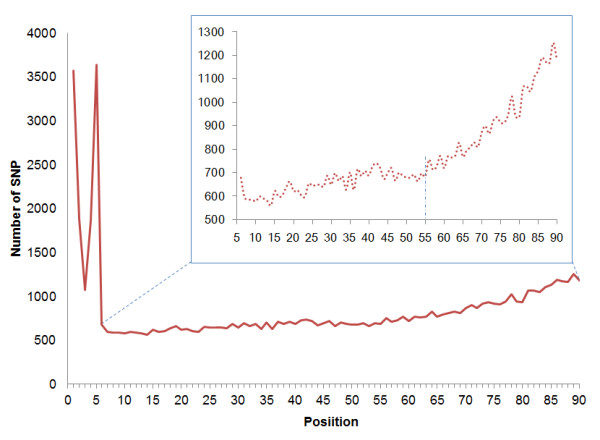
**Density distribution of FNR loci along the reads**. The density distribution of false negative SNPs was calculated and plotted. A large proportion of false negative SNPs located in the first five bases of each read, indicating great influence of disruption of recognition site. The inset shows the magnified distribution from position 6 to 90 along read and the dashed vertical line represents the position 55 after which the number of false negative loci increased sharply.

To further evaluate the SNP typing accuracy, we used the genotyping data generated by Illumina 1 M BeadArray to determine the concordance (Table 5). Of 108,735 SNPs which should be on target regions of RRL, 90.33% (98,220 SNPs) were genotyped with high confidence. In total, 98.19% of genotyping alleles were consistent with each other. The concordance rate of homozygote loci was over 99.8%, and that of heterozygote was only 92.56%. Among 1,708 inconsistent heterozygotes, over 99.7% loci were scored as homozygotes because of under calling. The low concordance rate was mainly due to low depth and uneven depth distribution of sequencing reads. As we performed *in silico *digestion and SNP calling using *hg18 *as reference, some bases in the enzyme recognition sites may be candidate SNPs in YH genome. If these loci were homozygous which were different from the reference, the corresponding target region could be excluded from the final RRL and the corresponding loci were accordingly undetectable, so that it may contribute to FNR (see discussion and Figure 3). In the same way, these heterozygous SNPs will lead to one corresponding allele missed and wrong genotyping results, according to statistical analysis, 971 (57.12%) loci were due to these SNPs located in enzyme recognition sites.

**Table 5:** Comparison of RRL sequencing and Illumina Beadchip genotyping results

					*Tsp *45I RRL sequencing		
	
		Concordance			Discordance		
	
			**HOM ref**.	**HOM mut**.	**HET ref**.	**HET mut**.	Total
Illumina genotyping	HOM ref.	55,435(99.95%)	---	5	22	0	27
	HOM mut.	19,847(99.80%)	21	1	18	0	40
	HET ref.	21,244(92.56%)	1458	245	3	2	1708
	HETmut.	0(0.00%)	4	0	0	0	4
	Total	96,445(98.19%)	1483	251	43	2	1779

The alleles genotyped by Illumina platform and RRL sequencing were classified into four categories: HOM ref. (homozygotes where both alleles are identical to the reference), HOM mut. (homozygotes where both alleles differ from the reference), HET ref. (heterozygotes where only one allele is identical to the reference), and HET mut. (heterozygotes where both alleles differ from the reference and also differ from one another).

### Genome coverage and MAF distribution

**Figure 4:**
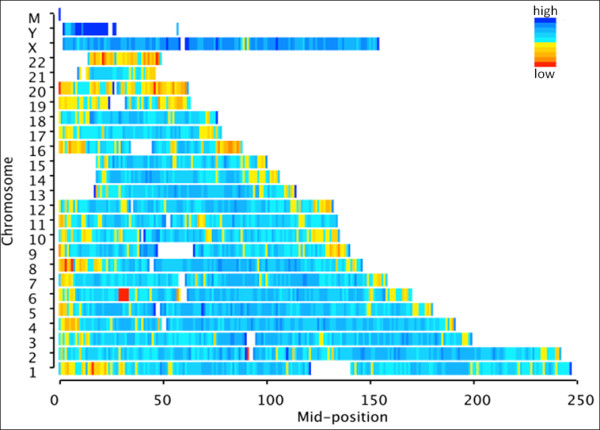
**Distribution of putative SNPs along chromosomes of reference genome**. The x-axis represents the relative position across each chromosome, and the y-axis represents chromosome coordinates of the reference genome. The colour from red to blue indicates the increased density of putative SNPs in each selected window across the chromosome.

Although a number of SNPs can be identified by RRL method, they are different from the other high throughput genotyping platforms such as Illumina Human 1 M Duo_v3.B chip which can genotype about one million SNPs selected preferentially with the purpose of providing good coverage and distribution. To evaluate the genome coverage of RRL method, we performed comparison between RRL and Illumina 1 M chip, our results indicated that RRL provided overall lower coverage in all three populations. Among nine enzymes, the coverage provided by *Tsp *45I was 60% in CHB+JPT populations at r^2 ^> 0.8, 78% by *Mbo II *with the demand to cover 2.3 M SNPs which would double the cost for detection. The best coverage provided by Human 1 M chip in CEU and CHB+JPT HapMap populations was 95% and 93%, respectively, and 76% in YRI population (Additional file 1). We also investigated the distribution of SNPs across the whole genome by *Tsp *45I RRL (Figure 4), the density of SNPs in each chromosome was totally even except for chromosome 20, chromosome 22 and some regions near telomere. The commercial products provided better coverage owing to the preferential selection of tag SNPs, but preferential selection made MAF distribution skew to the common SNPs (MAF > 0.1). The MAF distribution in RRL method, Illumina 1 M chip and HapMap populations were plotted, demonstrating the real MAF distribution of RRL method in populations without any bias (Additional file 2).

## Discussion

SNPs are the most abundant markers that are evenly distributed throughout genome. In human genome over 30 M SNPs are identified by HapMap Project [1,2] and 1000-genomes Project [3]. In this study we comprehensively accessed Restriction Enzyme-based RRL method with YH genome, which was the first complete Asian genome, including over 30 × whole genome sequencing data.

Compared with YH genome we observed high FDR and FNR of SNP calling, however, our further analysis found that 78.65% SNPs were already present in the dbSNP database, implying that these loci were possibly newly identified by RRL method. Repeat sequences and base quality contributed a great portion: 84.2% of FDR loci were confirmed to locate on repeat regions. We assumed to some extent that it was due to the different sequencing platform and sequencing strategy, YH WGS was performed on the Illumina Genome Analyzer that generated average 35 bp length reads obviously shorter than RRL sequencing performed on Illumina Hiseq 2000 with average read length of 90 bp. It was consistent with the fact that longer read length could improve the accuracy of mapping into reference and consequent lower FDR; Meanwhile, longer read length also leaded to lower quality score and consequent higher FDR, therefore we set up highly stringent filter parameter to reduce the FDR. We concluded that stringent filter criteria and repeat masking were necessary for increasing the accuracy of SNP calling.

FNR is another important parameter to access the power of this method, and due to lack of large-scale validation approach, published reports seldom include the evaluation of FNR. The error ratio of sequencing increases with read length, which may be one of the contributors for high FNR. Generally speaking, poor quality score of sequencing would not increase FNR, but FDR; however the major cause of the FNR in our study was due to the low quality score of sequencing. We consider false discovery worse than false negative in our RRL method, to confine the FDR well, we set relatively stringent parameters of SNP calling, which leaded to the lost of SNPs with low quality score as a consequence. Given restriction enzyme-based RRL methods always generate the fragments from the same start and end position, it formed low quality blocks at the 3'-end of enzyme fragments. The density distribution of FNR loci from six to ninety base along read indicated the number of FNR loci increased with position nearer to the end of reads (Figure 3) and significant increment of FNR was observed after 55 sequencing cycles. Moreover, part of false negative loci occurred in first five positions corresponding to enzyme recognition site because all of these candidate SNPs located in recognition site would be missed by RE digestion-based methods. From the comparison with genotyping results from Illumina 1 M Beadchip, the concordance rate of heterozygotes loci was only 92%, extremely lower than that of homozygotes. Over 99.7% of inconsistent heterozygotes were under-called to homozygotes partially because of low quality and inadequate depth. Moreover, 57.1% of these under-called loci were due to the disruption of recognition site by SNPs incorporation mentioned above. The discordance rate indicated the actual impact of disruption of recognition site. The disruption of the restriction site by a SNP can never totally be ruled out and merited careful attentions.

Given Restriction Enzyme-based RRL method identified SNPs around the restriction enzyme recognition site, only part of them was tag SNPs to represent a region of the genome with high LD. A strong correlation between the number of SNPs and coverage was observed in our simulative results. Clearly, there was a trade-off between SNPs density and genome coverage.

## Conclusions

RRL method combined with high-throughput sequencing is demonstrated to be an effective way for SNP discovery in individuals or populations. Our YH RRL data displayed high coverage and specificity of target region and identified over 257 K SNPs with about 8G sequencing data. Comprehensive evaluation with this method clarified the factors contributing to FDR and FNR of SNP identification and also presented the potential solution to improve the SNP calling accuracy. Our study extended the scope of this method and highlighted its application in the future.

## Authors' contributions

YD, HJ and CL designed and performed this study and drafted the manuscript. YC and QL performed the data analysis and helped to draft the manuscript. JW and YQ performed the library construction and sequencing. MZ participated in its design and coordination. XZ conceived, designed, and supervised the study. All authors read and approved the final manuscript.

## Supplementary Material

Additional file 1 **Summary of genomic coverage and MAF distribution of different enzymes**. This file shows statistics including number of markers, genomic coverage, MAF distribution and spacing when performing *in silico *digestion by nine restriction enzymes. Compared to the Human 1 M Duo_v3.B, *Tsp *45I is the most suitable enzyme considering both marker density and the other parameters. CEU: the HapMap data on individuals of European ancestry (http://www.hapmap.org, phase 2, HapMap-CEU); CHB: the HapMap data on individuals of Chinese ancestry (http://www.hapmap.org, phase 2, HapMap-CHB); JPT: the HapMap data on individuals of Japanese ancestry (http://www.hapmap.org, phase 2, HapMap-JPT); YRI: the HapMap data on individuals of Yoruba ancestry (http://www.hapmap.org, phase 2, HapMap-YRI). Click here for file

Additional file 2 **MAF distribution of putative SNPs in *Tsp *45I RRL and Illumina 1 M Beadchip**. The MAF distribution of putative SNPs in *Tsp *45I RRL was coincided with the curve of HapMap release24 data, but the distribution of SNPs on Illumina 1 M Chip was distinctly biased toward common SNPs. Click here for file
