# Supplementary material for: Comprehensive evaluation of SNP identification with the Restriction Enzyme-based Reduced Representation Library (RRL) method
Source: BMC Genomics. 2012 Feb 16;13:77. doi: 10.1186/1471-2164-13-77 (PMC3305556; doi:10.1186/1471-2164-13-77)
Supplement: Additional file 1 — Summary of genomic coverage and MAF distribution of different enzymes. This file shows statistics including number of markers, genomic coverage, MAF distribution and spacing when performing in silico digestion by nine restriction enzymes. Compared to the Human 1 M Duo_v3.B, Tsp 45I is the most suitable enzyme considering both marker density and the other parameters. CEU: the HapMap data on individuals of European ancestry (http://www.hapmap.org, phase 2, HapMap-CEU); CHB: the HapMap data on individuals of Chinese ancestry (http://www.hapmap.org, phase 2, HapMap-CHB); JPT: the HapMap data on individuals of Japanese ancestry (http://www.hapmap.org, phase 2, HapMap-JPT); YRI: the HapMap data on individuals of Yoruba ancestry (http://www.hapmap.org, phase 2, HapMap-YRI). [file 1471-2164-13-77-S1.PDF]

Types	Human1M Duo_v3.B	*Tsp* 45I	*Ava* I	*Sac* I	*Hind* III	*Pvu* II	*Sfc* I	*Dra* I	*Bcc* I	*Mbo* II
Number of Markers per sample	1,199,187	1,074,049	59,204	48,250	79,308	137,942	319,623	774,892	1,750,903	2,298,087
Genomic Coverage ($r^2 \geq 0.8$)										
CEU	0.95	0.57	0.02	0.05	0.11	0.13	0.26	0.49	0.67	0.77
CHB+JPT	0.93	0.59	0.02	0.05	0.11	0.14	0.27	0.50	0.68	0.78
YRI	0.76	0.36	0.01	0.02	0.05	0.06	0.13	0.30	0.45	0.58
Minor Allele Frequency (Mean / Median)										
CEU	0.2/0.18	0.15/0.10	0.15/0.10	0.15/0.10	0.15/0.10	0.15/0.10	0.15/0.10	0.15/0.10	0.15/0.10	0.15/0.10
CHB+JPT	0.18/0.17	0.14/0.07	0.14/0.07	0.14/0.08	0.14/0.07	0.14/0.07	0. 14/0.07	0.14/0.06	0.14/0.07	0.14/0.07
YRI	0.20/0.17	0.16/0.12	0.16/0.12	0.16/0.13	0.16/0.12	0. 16/0.12	0.16/0.12	0.16/0.12	0.16/0.12	0.16/0.12
Spacing (kb)										
(Mean / Median)	2.4/1.5	2.8/0.6	50.6/1.0	62.1/9.7	37.7/8.5	21.7/3.1	9.3/1.4	3.8/0.7	1.7/0.5	1.3/0.5

Additional file 1 Summary of genomic coverage and MAF distribution of RRL using different enzymes. This file shows some statistics including number of markers, genomic coverage, MAF distribution and spacing when performing *in silico* digestion using nine restriction enzymes. Compared to the Human 1M Duo_v3.B, *Tsp* 45I should be the most suitable choice on account of the tradeoff between the marker density and other parameters. CEU: the HapMap data on individuals of European ancestry (http://www.hapmap.org, phase 2, HapMap-CEU);

CHB: the HapMap data on individuals of Chinese ancestry (http://www.hapmap.org, phase 2, HapMap-CHB); JPT: the HapMap data on individuals of Japanese ancestry (http://www.hapmap.org, phase 2, HapMap-JPT); YRI: the HapMap data on individuals of Yoruba ancestry (http://www.hapmap.org, phase 2, HapMap-YRI).
