# Supplementary material for: Comprehensive evaluation of SNP identification with the Restriction Enzyme-based Reduced Representation Library (RRL) method
Source: BMC Genomics. 2012 Feb 16;13:77. doi: 10.1186/1471-2164-13-77 (PMC3305556; doi:10.1186/1471-2164-13-77)
Supplement: Additional file 2 — MAF distribution of putative SNPs in Tsp 45I RRL and Illumina 1 M Beadchip. The MAF distribution of putative SNPs in Tsp 45I RRL was coincided with the curve of HapMap release24 data, but the distribution of SNPs on Illumina 1 M Chip was distinctly biased toward common SNPs. [file 1471-2164-13-77-S2.PDF]

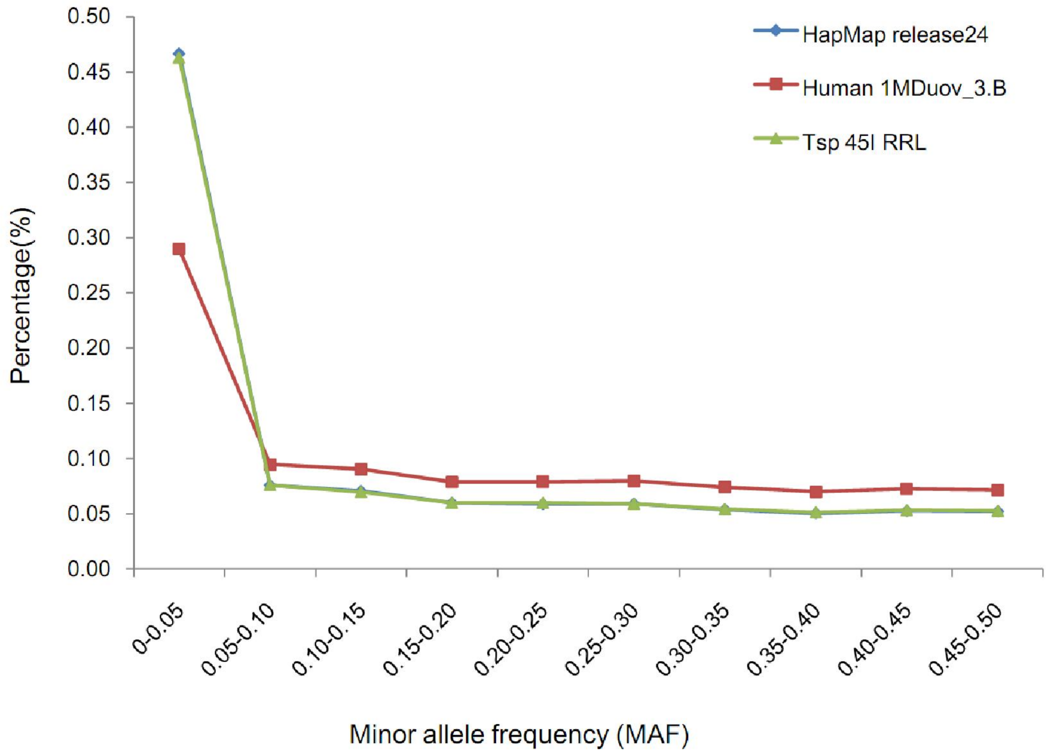

Additional file 2 MAF distribution of putative SNPs in *Tsp* 45I RRL and Illumina 1M Beadchip. MAF distribution of putative SNPs in *Tsp* 45I RRL was much closer to the real distribution in HapMap release24 data, but the Illumina 1M Beadchip was distinctly biased toward common SNPs.
